# The developmental origin of brain tumours: a cellular and molecular framework

**DOI:** 10.1242/dev.162693

**Published:** 2018-05-14

**Authors:** Roberta Azzarelli, Benjamin D. Simons, Anna Philpott

**Affiliations:** 1 Department of Oncology, University of Cambridge, Hutchison/MRC Research Centre, Cambridge Biomedical Campus, Cambridge CB2 0XZ, UK; 2 Wellcome Trust Centre for Stem Cell Research, University of Cambridge, Tennis Court Road, Cambridge CB2 1QR, UK; 3 The Wellcome Trust/Cancer Research UK Gurdon Institute, University of Cambridge, Tennis Court Road, Cambridge CB2 1QN, UK; 4 Cavendish Laboratory, Department of Physics, University of Cambridge, JJ Thomson Avenue, Cambridge CB3 0HE, UK

## Abstract

The development of the nervous system relies on the coordinated regulation of stem cell self-renewal and differentiation. The discovery that brain tumours contain a subpopulation of cells with stem/progenitor characteristics that are capable of sustaining tumour growth has emphasized the importance of understanding the cellular dynamics and the molecular pathways regulating neural stem cell behaviour. By focusing on recent work on glioma and medulloblastoma, we review how lineage tracing contributed to dissecting the embryonic origin of brain tumours and how lineage-specific mechanisms that regulate stem cell behaviour in the embryo may be subverted in cancer to achieve uncontrolled proliferation and suppression of differentiation.

## Abstract (precis)

**Summary:** Lineage-tracing work in glioma and medulloblastoma reveals similarities between neuronal development and brain tumours, identifying potential new therapeutic avenues that exploit vulnerabilities in tumour growth patterns.

## Introduction

Neurological cancers are among the most feared malignancies. They include medulloblastoma, the most common malignant brain tumour in children, and high-grade glioblastoma, one of the most lethal adult cancers (Table 1) (Louis et al., 2016). Treatment for medulloblastoma requires high dose multi-modal chemotherapy and radiotherapy that come with significant and long-term adverse consequences, even when a cure is obtained, whereas glioblastoma is almost invariably fatal even after treatment. Hence, there is a pressing need to understand more about the biology of these diseases, so that therapy can be effectively targeted to the malignant cells and not to the surrounding tissue.

**Table 1.**
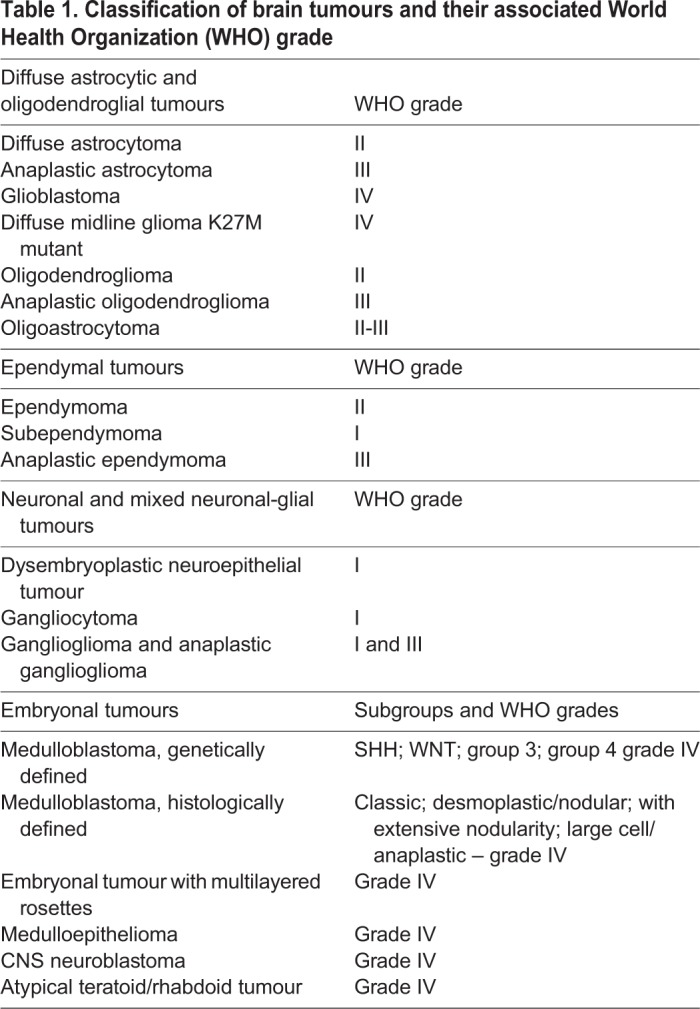
**Classification of brain tumours and their associated World Health Organization (WHO) grade**

For many years, research has focussed on what different types of neurological tumours have in common with other malignancies and with each other, e.g. the disruption of classic oncogenic and tumour suppressor pathways, but this approach has had little effect on improving survival rates. More promising perhaps is the emerging consensus that brain tumours are maintained by a specific neural or glial cancer ‘stem cell-like’ population that self-renews and gives rise to differentiated progeny (Galli et al., 2004; Singh et al., 2003, 2004; Vescovi et al., 2006). Whether tumours initiate in stem cell-like populations or arise from progenitors that, through mutation, acquire stem cell-like potential remains unknown. Moreover, cancer stem cells and their progeny can demonstrate considerable plasticity (Batlle and Clevers, 2017), and brain tumours that arise from them often harbour mixed cell populations that are very reminiscent of normal developing brain tissue (Lan et al., 2017; Pollen et al., 2015; Tirosh et al., 2016).

**Fig. 1.**
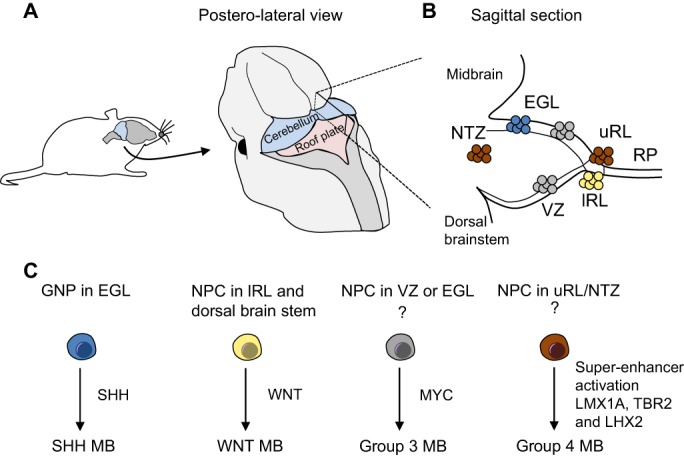
**Cell of origin in medulloblastoma subgroups.** (A) Posterolateral view of the mouse developing cerebellum. (B) Sagittal section of the developing cerebellum showing the location of the precursors that give rise to the distinct medulloblastoma subgroups shown in C. Sonic hedgehog-positive (SHH) medulloblastomas derive from GNPs in the EGL (blue), WNT-positive medulloblastomas derive from the lower RL and dorsal brain stem (yellow), group 3 medulloblastomas are thought to originate from either VZ or EGL progenitors overexpressing the oncogene Myc (grey) and group 4 medulloblastomas have been proposed to derive from cells with active LMX1A, TBR2 and LHX2 super-enhancers in the NTZ that contains deep nuclei originating from the upper RL (brown). Question marks under the cell of origin in groups 3 and 4 highlight the difficulty in pinpointing a specific cell of origin for these subgroups. Medulloblastoma classification is also constantly evolving and further subdivisions within these four subgroups have been recently reported (see Cavalli et al., 2017). EGL, external granule cell layer; GNPs, granule neuron precursors; lRL, lower rhombic lip; MB, medulloblastoma; NTZ, nuclear transitory zone; RP, roof plate; uRL, upper rhombic lip; VZ, ventricular zone.

The possibility that neurological cancers are ‘locked in’ to a developmental programme and may retain many of the controls that impinge on these cell populations during development opens up new and exciting opportunities for understanding and targeting these cancers. Some of these opportunities are already being exploited in the treatment of paediatric neurological malignancies, where the relationship of cancer cells to spatially and temporally distinct embryonic precursors is better understood (Cavalli et al., 2017; Phoenix et al., 2012; Ramaswamy et al., 2016). For example, medulloblastoma can be classified into distinct subgroups depending on histological features and genetic profiling, and it has become clear over the years that differences in these subgroups may relate to their origin within different regions of the cerebellum (Fig. 1) (Bihannic and Ayrault, 2016; Cavalli et al., 2017; Gibson et al., 2010; Li et al., 2013; Phoenix et al., 2012). This classification has the potential to profoundly influence future research and treatment. In particular, it identifies subgroups of patients with different prognoses and sensitivity to drugs, which has already influenced therapeutic intervention strategies in some children (Ramaswamy et al., 2016).

In this Review, we will consider both paediatric and adult central nervous system tumours through the lens of the developmental biology paradigms that they exploit to maintain a stem/progenitor identity, while at the same time producing both proliferating and differentiating progeny. We will also discuss the extent to which viewing these cancers as diseases of development might reveal new therapeutic approaches, exploiting tissue-specific oncogenes and the aberrant developmental phenotype while sparing normal tissue.

## The search for brain tumour stem cells

The stem cell hypothesis of tumour maintenance has become increasingly prominent in recent years (Batlle and Clevers, 2017). In this paradigm, bulk tumours are fed by a subpopulation of slow-cycling stem cell-like cells that harbour tumour-initiating potential. Cancer stem cells are generally thought to be resistant to treatment, yet retain the ability to reconstitute the varied cell types within the heterogeneous tumour mass once treatment ceases. Brain tumours were among the first cancers in which stem cell-like cells were identified and isolated *in vitro*, although how this behaviour relates to their *in vivo* role remains somewhat unclear (Galli et al., 2004; Singh et al., 2003, 2004). A subpopulation of CD133^+^ cells was isolated from paediatric human brain tumours that exhibited stem cell-like properties *in vitro* and that, when injected *in vivo*, recapitulated features of the original tumour, including its heterogeneous cell composition (Singh et al., 2004). Similarly, cells with stem-like properties have been isolated from a wide range of paediatric tumours, such as glioma, medulloblastoma, primitive neuroectodermal tumours and ependymoma (Galli et al., 2004; Hemmati et al., 2003). In common with non-malignant neural precursor cells, these tumour cells can be grown *in vitro*, allowing a direct comparison between normal and tumour stem cells, and facilitating the identification of drugs that may selectively act on cancer cells and not their normal counterparts (Bressan et al., 2017; Pollard et al., 2009).

### Tumour cell of origin: stem, progenitor or differentiated cell types?

Brain tumours can arise from stem, progenitor and/or more mature cells, and one might expect their cell of origin to significantly influence subsequent cell behaviour. Understanding the cell of origin of each tumour type may also expose lineage-specific therapeutic vulnerabilities and/or opportunities to identify early malignant or even pre-malignant abnormal cell states. Some cells may certainly be more vulnerable to oncogenic assault than others. Although functional studies provide strong evidence for stem cell-like behaviour in certain subpopulations of brain tumours, the identification of definitive cell surface markers of these cells has been challenging. For example, while CD133-positive cells have been shown to harbour tumour-initiating potential, so too have CD133-negative cells (Beier et al., 2007; Ogden et al., 2008; Read et al., 2009). The cell surface marker CD15 (also known as stage-specific embryonic antigen, SSEA1) has been proposed as a general marker for brain tumour stem cells in both gliomas and medulloblastomas (Son et al., 2009; Ward et al., 2009). However, the studies that implicate CD15 have indicated that the ability to maintain tumours may not reside solely in stem-like cells, but may extend to cells with a more restricted progenitor-like identity. For example, a rare population of CD15-positive cells identified in human medulloblastomas (Read et al., 2009) and from a *Ptch1^+/−^* medulloblastoma mouse model (Read et al., 2009; Ward et al., 2009) also express ATOH1 (also known as MATH1), and so resemble granule neuron precursors rather than stem cells. Thus, although stem cells are thought to reside at the apex of a hierarchy that maintains tumour growth, several lines of evidence indicate that actively cycling fate-restricted progenitors might also contribute to the formation and progression of tumour masses (Vanner et al., 2014). Indeed, lineage-tracing studies have demonstrated that type I SHH-driven medullobastomas can be initiated from Atoh1-positive granule neuron precursors (Schüller et al., 2008; Yang et al., 2008), whereas oligodendrogliomas, which represent 5-20% of all gliomas, mainly originate from NG2-positive oligodendrocyte precursor cells (Liu et al., 2011; Persson et al., 2010). Intriguingly, in different mouse models of gliomagenesis, tumour growth potential has been shown not to correlate directly with self-renewal, and it is instead the non self-renewing lineages that generate tumours more rapidly and with higher penetrance (Barrett et al., 2012). Such behaviour could reveal a requirement for lineage-restricted pathways for initiating or maintaining tumours.

Our ability to distinguish cell types in the brain allows us to compare the tumourigenic potential of specific neural stem and progenitor populations. For example, activation of oncogenes, such as KRas^G12D^, or inactivation of tumour suppressors, such as p53, Rb, PTEN, Arf or Nf1, has been used to directly address the tumourigenic potential of different cells. These works reveal that neural stem cells (NSCs) and progenitor cells are more readily transformed than differentiated cell types, and embryonic radial glia cells (RGCs) are more prone to transformation than postnatal stem cells (Alcantara Llaguno et al., 2009; Jacques et al., 2010; Munoz et al., 2013). Moreover, evidence points to astrocytes and oligodendocyte progenitors as also having the potential to act as the cells of origin in gliomas (Zong et al., 2015). As the majority of astrocytomas are preferentially located in areas rich in neural progenitor cells (Chow et al., 2011; Zong et al., 2015), the tumour-initiating capacity of astrocytes has been difficult to assess, as they co-express markers of neural precursor cells (e.g. GFAP). However, with over 20% of astrocytomas formed in non-proliferative zones, it follows that either GFAP-positive NSCs have migrated to distant sites, or that tumours originate from mature astrocytes (Chow et al., 2011). Multiple lines of evidence also support a role for NG2-positive oligodendrocyte precursor cell transformation in glioma (Liu et al., 2011; Persson et al., 2010), including histopathological expression of oligodendrocyte precursor cell markers in human samples, overlap between the molecular signature of proneural subtype glioma and that of oligodendrocyte precursor cells, and the great expansion of oligodendrocyte precursor cells in comparison with NSCs, astrocytes or neurons upon tumour suppressor gene inactivation and prior to malignant transformation.

The identification of the cell-of-origin in medulloblastoma has been even more challenging because of the high degree of inter-tumoural heterogeneity. Medulloblastomas can be classified into discrete subgroups based on gene expression and tumour-driving mutations (Gibson et al., 2010) (Fig. 1). Importantly, different tumour subgroups arise from different cell types in distinct locations and are hence likely to arise from different tumour-initiating populations: group 1 medulloblstoma is SHH driven and originates from granule neuron precursors in the cerebellar external granule cell layer (Schüller et al., 2008; Yang et al., 2008); group 2 medulloblastoma is WNT driven and arises from progenitors in the dorsal brain stem (Phoenix et al., 2016); group 3 medulloblastoma is associated with Myc overexpression in granule neuron precursors, ventricular zone stem cells or in other classes of progenitors (Kawauchi et al., 2012; Pei et al., 2012; Wang and Wechsler-Reya, 2014); and group 4 is thought to originate from deep nuclei precursors located in the upper rhombic lip (Lin et al., 2016) (Fig. 1B,C). Further refinement of these four groups into 12 distinct subtypes through the combination of genome-wide DNA methylation, gene expression and pathway analysis revealed that the SHH group of tumours could be divided into four subtypes: α, β, γ and δ (Cavalli et al., 2017). These subtypes correlate with differing prognoses, and SHH γ-subtype children who have excellent survival rates could thus be included in the group of young patients treated with reduced radiation in the future (Bavle and Parsons, 2017; Cavalli et al., 2017). The classification of medulloblastoma based on the tumour cell of origin, with an emphasis on understanding the differences in the ensuing pathogenesis of disease, is probably the best example of a tumour type where such an understanding could inform therapeutic regimens for patient benefit.

Overall, it is clear that brain tumours arise from multiple cell types that are distinguishable both by location and by degree of differentiation, although the precise cell of origin is rarely clear. Morphologically similar cancers may yet be shown to arise from different cell populations. Moreover, mature brain cells are not completely resistant to oncogene-mediated transformation, as astrocytes and even mature neurons can dedifferentiate to form gliomas in mice (Friedmann-Morvinski et al., 2012). However, the general principle seems to hold that the more mature a cell is, the more resistant it is to transformation.

### Mechanisms by which stem cell-like phenotypes are acquired

**Fig. 2.**
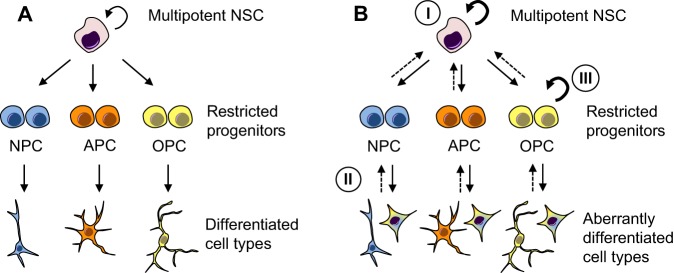
**Mechanisms of ‘stemness’ acquisition in cancer.** (A) Under physiological conditions, multipotent neural stem cells (NSCs) self-renew and differentiate into fate-restricted progenitors, which are capable of lineage amplification and differentiation to the three main cell types in the brain: neurons (blue), astrocytes (orange) and oligodendrocytes (yellow). (B) Cancer cells can arise from de-regulation of NSC self-renewal (curved bold arrow, I), from de-differentiation of cells that revert back to a stem or progenitor-like state (dashed arrows, II) and/or from failed differentiation of stem (I) and progenitor (III) cells that are locked in a pro-proliferative state and differentiate aberrantly (curved bold arrows, I and III). APC, astrocytic progenitor cells; NPC, neural progenitor cells; OPC, oligodendrocyte precursor cells.

While tumours can arise from progenitor populations, the extent to which they must revert to a stem cell-like state to overcome restrictions in progenitor cell proliferation as well as lineage restriction is an area of debate. It is clear that progenitors can initiate tumour growth in medulloblastoma and glioma [granule neuron precursors in medulloblastoma and oligodendrocyte precursor cells in gliomas (Liu et al., 2011; Persson et al., 2010; Schüller et al., 2008; Yang et al., 2008)], while these cells give rise to progeny that can differentiate into both to glia and neurons, a property of multipotent stem cells. Oncogenic mutations could be present in multipotent stem cells, but only result in tumourigenic potential when cells adopt a more restricted progenitor cell identity, suggesting an interaction between the particular mutation and the developmental programme (Fig. 2). In addition, fate-restricted progenitors could reacquire stem cell-like properties through a process of dedifferentiation, providing them with the plasticity necessary to differentiate into multiple lineages. The ability to revert back to a tumourigenic glioma stem cell-like state can be achieved by forced oncogene expression in differentiated glioblastoma cells (Suvà et al., 2014) and even in mature post-mitotic neurons and astrocytes (Friedmann-Morvinski and Verma, 2014) (Fig. 2). This suggests a potential contribution of environmental factors and cell cycle re-entry during the course of reversion. However, the interplay between environmental factors and oncogenes, and the impact of dedifferentiation and dysregulation of cell fate on cancer formation have only recently been proposed for some epithelial tumours (Krah and Murtaugh, 2016) and are not well defined for brain tumours.

In addition to dedifferentiation, an active impediment to differentiation could also ‘trap’ cells in a proliferative pro-tumourigenic state (Fig. 2). Mechanisms of differentiation failure have been the subject of intensive studies as the unlocking of any latent ability to differentiate could be exploited as a therapeutic strategy to drive neural and glial tumour cells into a permanently post-mitotic state (Fortier et al., 2010; Hu et al., 2013). Proof of principle of this therapeutic approach is provided by acute promyelocytic leukaemia in which differentiation therapy (all-trans-retinoic acid/arsenic trioxide) negatively impacts proliferative potential, extinguishes self-renewal and subsequently increases survival from 10% to over 90% (de The and Chen, 2010). However, attempts to drive glioblastoma cells into terminal differentiation have, so far, been inconsistent (Carén et al., 2015; Park et al., 2017; Piccirillo and Vescovi, 2006).

Although much evidence points to recapitulation of developmental programmes in the behaviour of brain cancer cells, these could also be undergoing an aberrant regenerative process that, in itself, could exploit mechanisms originally active in adult or embryonic NSCs. This is an exciting and yet poorly investigated topic and future research should focus on understanding the similarities between embryonic neural stem/progenitor cells, adult neural stem/progenitor cells and injury-reactivated cells, and investigate the potential contribution of regenerative responses to brain tumour formation (Torper and Götz, 2017; Urbán and Guillemot, 2014).

## Tracing the lineage progression of brain tumour cells

Although the highly proliferative capacity of fate-restricted progenitors plays a significant role in tumour growth and progression, therapies that target proliferation have drastically failed, mainly because the resident slow-cycling stem-like cells can become reactivated and cause tumour relapse (Hambardzumyan et al., 2008; Vanner et al., 2014). To develop successful therapies, it is thus essential that we understand not only the tumour cell of origin and how it can acquire a stem cell signature, but also lineage progression within the tumour. Efforts to study the fate of cells and to establish whether aspects of a normal hierarchical lineage progression are conserved during tumourigenesis have focused on quantitative lineage-tracing assays. To this end, different studies have used diverse and complementary approaches, such as analysis of mutational landscape data, single-cell RNA sequencing, clonal size distribution and quantitative statistical modelling to investigate lineage progression and clonal evolution in medulloblastoma, oligodendroglioma and glioblastoma.

Genomic analysis of individual human medulloblastomas immediately post-diagnosis and after therapy showed that fewer than 12% of diagnostic genetic events were present in the relapsed tumour sample. Indeed, the genetic clone seen to dominate the tumour was different before and after therapy; close analysis revealed that the dominant clone in relapsed tumours arose from a previous minor clone that was, nevertheless, present at initial diagnosis (Morrissy et al., 2016). Similar results were obtained in glioblastoma, in which recurrent tumours are thought to be seeded by cells derived from the initial tumour at a very early stage of their evolution (Johnson et al., 2014). This type of pattern would suggest that genetic variations play a role in clonal evolution at recurrence or after therapy. However more recent work has partially challenged this view. Through single-cell RNA sequencing, Suvà and colleagues have identified a hierarchical architecture in oligodendroglioma reminiscent of a developmental programme, with evidence for an undifferentiated compartment that shares a gene expression signature with neural stem and progenitor cells, fuels tumour growth and transitions into differentiation along the two glial lineages: astrocytes and oliogodendrocytes (Tirosh et al., 2016; Venteicher et al., 2017). Importantly, the authors suggest that this hierarchy is anchored in a developmental programme and has not evolved through genetic evolution, which could otherwise modulate the patterns of tumour cell self-renewal and differentiation. However, as the oligodendroglioma could not be expanded through xenotransplantation, a complete phylogenetic reconstruction was missing and genetic influences can not be entirely ruled out.

In a similar vein, new findings have been reported on tumour cell dynamics in glioblastoma using a novel clonal fate mapping approach based on genetic barcoding previously applied to mammary tumour models (Lan et al., 2017; Nguyen et al., 2015). By combining DNA barcoding of primary human glioblastoma cells with quantitative analysis of clone size following serial xenotransplantation into mouse, Lan et al. have shown that the observed heterogeneity in clonal expansion is not associated with variability in the mutational landscape, but derives from stochastic fate decisions of tumour cells obtained within a conserved developmental-like hierarchy (Lan et al., 2017). In this model, tumour expansion is driven by a subpopulation of slow-cycling stem-like cells that renew while giving rise to a rapidly cycling intermediate progenitor-like population, which self-renew and generate short-lived non-dividing progeny. Interestingly, cells isolated from primary glioblastoma have been shown to have a transcriptional signature reminiscent of outer radial glia cells (Patel et al., 2014; Pollen et al., 2015), a self-renewing developmental precursor located in the basal regions of the human cerebral cortex (Fietz et al., 2010; Hansen et al., 2010). This novel finding suggests that similar mechanisms might regulate expansion and self-renewal of tumour cells and of these normal developmental precursors. This behaviour, which is highly reminiscent of lineage progression during neural development, has also been proposed for SHH-driven medulloblastoma. In the Ptch1 heterozygous mouse model of medulloblastoma, studies of proliferation kinetics and genetic lineage tracing have shown that slow-cycling Sox2-positive stem-like cells at the apex of a hierarchy give rise to highly proliferative intermediates (marked by Dcx and Ki67 expression) that differentiate into NeuN-positive neurons, which then undergo rapid apoptosis (Vanner et al., 2014). Whether the clonal dynamics of tumour growth in medulloblastoma reflect that inferred from the dynamics of tumour cells in glioblastoma remains unknown.

The studies discussed in this section not only support the existence of seemingly conserved lineage hierarchies in brain tumours that are reminiscent of a normal developmental programme, but also shed light on the relative contribution of genetic variation and developmental mechanisms to inter- and intra-tumoural heterogeneity. This is an important area of study, as resolution of the clonal dynamics and lineage progression of neurological tumours could provide novel approaches to therapy. As shown in the context of other epithelial tumours (Driessens et al., 2012; Sánchez-Danés et al., 2016; Alcolea et al., 2014; Frede et al., 2016), brain tumour growth could also rely on the preferential loss of differentiating divisions, leading to a bias in cell fate decision towards dividing daughter cells. Thus, manipulating this balance to alter cell fate decisions, rather than inhibition of cell cycle, might prove to be a more effective therapeutic approach. Another benefit of these types of clonal analyses is that they may also reveal different types of clonal behaviour characterized by differential sensitivity to drugs. This has been shown, for example, in the mouse xenograft study of human glioblastoma, which is seen to contain two types of clones. A subpopulation of expanding clones, which depart from the behaviour of the bulk population, become selected for during temozolomide treatment, but are instead sensitive to the menin-MLL inhibitor, an epigenetic drug previously shown to be effective in H3.3 mutant paediatric glioblastoma (Lan et al., 2017). In summary, dynamic analysis of lineage progression, in combination with quantitative clonal analysis and genome-wide DNA and RNA sequencing, can provide a useful framework for developing effective combinatorial therapies.

## Epigenetic regulation of tumourigenesis

Although some oncogenic events are shared across multiple tumour types, distinct genetic lesions associated with specific types of tumours point to intrinsic differences in the way cells of different lineages respond to oncogenic assault. Moreover, lineage-specific transcriptional regulators have also been identified as context-dependent oncogenes and/or tumour suppressor genes, reinforcing the idea that key genes that regulate normal developmental lineages may become deregulated in cancer, subverting their normal function and resulting in uncontrolled proliferation and suppression of terminal differentiation (Garraway and Sellers, 2006; Vias et al., 2008). Such lineage-specific oncogenic function is likely to rely on the cell type-specific epigenetic environment in which oncogenic activation occurs, and to intersect with tissue-specific self-renewal and differentiation signalling pathways.

### The contribution of chromatin dysregulation to neurological cancers

In eukaryotes, DNA is wound around a core of nucleosomal histone proteins to form chromatin. Chromatin organization is of fundamental importance in the establishment and maintenance of cell-type specific transcriptional programmes during development and differentiation, and imposes the environment in which tissue-specific transcriptional regulators must act. Not surprisingly, alterations to the chromatin landscape can profoundly impact cell fate decisions in development and cancer. At its simplest level, chromatin remodelling is achieved through the concerted activity of proteins and enzymes that regulate histone methylation, histone acetylation, DNA methylation, and nucleosome tri-dimensional structure and repositioning (Jones et al., 2013). The importance of chromatin regulators in various types of cancer is highlighted by the recurrent copy number alterations or mutations at chromatin-modifying genes. Importantly, certain types of chromatin-modifying alterations are restricted to specific subgroups of tumours, as has been shown in medulloblastoma (Northcott et al., 2017; Robinson et al., 2012), and might thus impart lineage-specific vulnerabilities to distinct types of tumour cells. Understanding these vulnerabilities may provide insights into novel therapeutic approaches, and indeed many novel agents targeting chromatin modifiers are currently in development or in early clinical trials.

### H3.3 variants in paediatric gliomas

Diffuse intrinsic pontine gliomas (DIPGs, now included in the diffuse midline glioma classification – see Table 1) result in a median survival of only 9 months. Studies of this devastating malignancy have demonstrated that paediatric and adult gliomas are biologically and molecularly distinct. The most prominent difference lies in hotspot mutations in the gene encoding histone 3.3 variants, with only 0.2% of adult patients, yet 50% of paediatric patients, carrying these mutations (Schwartzentruber et al., 2012; Wu et al., 2012). The histone 3 variant 3 (H3.3) is cell-cycle independent and is incorporated into genic euchromatin regions or pericentromeric and telomeric regions by different associated proteins: *ATRX* and *DAXX*. Interestingly, in addition to mutations at key regulatory residues in histone H3.3, mutations have been reported in *ATRX* and *DAXX* (Huether et al., 2014; Schwartzentruber et al., 2012). The majority of DIPG patients carry a H3.3 Lys27Met (K27M) missense mutation, whereas a minority exhibit a H3.3 G34R/V mutation. Moreover, advances in genomic and bioinformatic techniques have allowed the sub-classification tumours based on common mutational patterns of histones. An analysis of a large dataset of around 1000 samples of paediatric and adult gliomas revealed that K27M and G34R/V H3.3 variants represent different biological subgroups (Mackay et al., 2017); K27M H3.3 tumours are found in 70% of DIPG and non-brainstem midline paediatric gliomas and exhibit selective mutations in CCND2 and TOP3A, whereas H3.3G34R/V-mutant tumours are restricted to the cerebral hemispheres and co-segregate with mutations in the histone-associated proteins ATRX and TP53. Methylation of H3.3 is reduced by the K27M mutation and this results in disrupted transcription (predominantly de-repression) of several cancer-associated genes (Bender et al., 2013; Chan et al., 2013). Overexpression of H3.3K27M, alongside other co-operating mutations, is required in the correct cell and, crucially, at the correct developmental time (in this case pre-natally) to generate a mouse model of paediatric high-grade glioma. This again demonstrates the importance of the spatiotemporal context in moving from an oncogenic assault to a full-blown tumour (Pathania et al., 2017).

### Control of methylation by Polycomb and Trithorax-group proteins

Methylation of both DNA and histones plays an important role in regulating gene expression levels. A recent genomic analysis across the different medulloblastoma subgroups revealed that group 3 and 4, in particular, carry somatic copy number aberrations and have transcriptional profiles that converge on deregulated methylation of H3K4 and H3K27 (Huether et al., 2014). Polycomb (PcG) and Trithorax (TrxG) protein complexes are responsible for epigenetic histone modifications that either repress or promote gene transcription, and several lines of evidence indicate that altered activity of these epigenetic modifiers may contribute to the neoplastic phenotype.

The methyltransferase EZH2, which is the enzymatic subunit of the polycomb repressive complex 2 (Prc2), is responsible for H3K27 trimethylation, a repressive mark that is tightly associated with inactive gene promoters. EZH2 is upregulated in various cancers, including medulloblastoma and glioblastoma, and it can act as a critical regulator of neoplastic proliferation, maintenance of stem cell-like features and inhibition of differentiation (Suvà et al., 2009; Vo et al., 2017). For example, small-molecule inhibition of EZH2 in glioblastoma and DIPG reduced tumourigenesis *in vivo* (Lan et al., 2017; Mohammad et al., 2017; Wang et al., 2017), whereas loss of EZH2 in medulloblastoma attenuated growth and promoted differentiation *in vitro* (Alimova et al., 2012). However, EZH2 inactivation in an *in vivo* mouse model of group 3 medulloblastoma resulted instead in accelerated tumour initiation and progression, due to de-repression of the proto-oncogene Gfi1, which cooperates with Myc (Vo et al., 2017). This reveals that EZH2 can act as both an oncogene and a tumour suppressor gene, depending on the context. Multiple other genes belonging to PRC1 and PRC2 complexes, including *BMI1*, *EED* and *SUZ12* have been found upregulated either in specific medulloblastoma subgroups or across medulloblastoma generally.

TrxG complexes sustain transcription via both their H3K4 methyltransferase activity and H3K27 demethylase activity that opposes PcG mediated repression, and components of the TrxG group of proteins have also been found to be mutated in high-grade gliomas and medulloblastoma (Huether et al., 2014). Moreover, the demethylase *KDM6A* (also called *UTX*) and the histone methyltransferases mixed lineage leukaemia, *MLL2* (*KMT2D*) and *MLL3* (*KMT2C*), display inactivating and truncating mutations, suggesting tumour suppressive functions. Interestingly, *KDM6A* and *MLL2* mutations have been found to be mutually exclusive, further reinforcing the likelihood that they regulate similar processes (Dubuc et al., 2013).

Mutations in isocitrate dehydrogenase 1 (IDH1), and less frequently in IDH2, occur in 80% of grade II and grade III astrocytomas and oligodendrogliomas, and are also found in high-grade glioblastomas that have arisen over time from these lower-grade gliomas (Staedtke et al., 2016). IDH mutations disrupt cellular metabolism. This ultimately leads to hypermethylation of histones and CpG islands, a so-called methylator phenotype, that brings about extensive dysregulation in gene expression (Turcan et al., 2012), which works in conjunction with additional mutations to drive tumourigenesis (Weller et al., 2015). Interestingly, progression to higher grade disease is often accompanied by overall decrease in methylation, but hypermethylation of a small subset of CpG islands associated with developmental regulators, including FOX, SOX and TBX family genes, which may ‘lock’ cells into a permanently self-renewing state (Bai et al., 2016). IDH activity and the pathways it regulates have therefore recently been proposed as a potentially important therapeutic targets in gliomas (Malta et al., 2017).

### Super-enhancer and bromodomain proteins

Recent excitement has accompanied the identification of enhancer regions where multiple transcriptional regulators are bound, and which direct a very high level of gene expression, so-called super-enhancers. Super-enhancers are thought to be essential for maintenance of cell identity (Hnisz et al., 2013), whereas aberrant super-enhancer formation and/or maintenance may underlie both inappropriate activation of oncogenic drivers and an alteration in cell fate and differentiation (Chipumuro et al., 2014; Lovén et al., 2013). Super-enhancers are characterised by very high levels of H3K27 acetylation. This leads to the accumulation of bromodomains and extra-terminal domain (BET) proteins, as well as more recruitment of the transcriptional cyclin-dependent kinase CDK7, which directs high levels of transcription (Larochelle et al., 2012; LeRoy et al., 2008; Rahman et al., 2011). Inhibition of BET or CDK7 has been used to target MYC-driven tumours in different contexts, as MYC expression in tumours is frequently maintained at a high level by an associated super-enhancer region (Sengupta and George, 2017). For example, BET inhibition in multiple myeloma cells and CDK7 inhibition in neuroblastoma cells led to preferential downregulation of super enhancer-associated genes, including MYC and other genes associated with the biology of the specific lineage of the tumour (Chipumuro et al., 2014; Lovén et al., 2013). Strikingly, these drugs show a remarkable selectivity for MYC-amplified cells. However, super-enhancer activity is also important in non MYC-driven tumours.

As described above, the majority of individuals with DIPG carry H3.3 mutations that are often accompanied by a reduction in the levels of PRC2-mediated H3K27 trimethylation. However, novel epigenetic analyses demonstrate that several genes not only retain H3K27 methylation but also showed increased H3K27 acetylation (Piunti et al., 2017), an epigenetic mark that is typically indicative of actively transcribed genes, and which correlates with BET protein association. Inhibition of BET proteins and of CDK7 has been used to successfully inhibit tumourigenesis in DIPG, preferentially disrupting transcription at super enhancer-associated genes. Many of the dysregulated genes are specifically involved in neuronal-lineage specification, including the bHLH factor ASCL1 (discussed below) (Nagaraja et al., 2017). Thus, super-enhancers can mediate transcriptional vulnerabilities that are specific to each tumour type and can point to previously unknown mechanisms of tumour pathobiology related to the lineage-specific transcriptional networks of the tumour cell of origin, illustrated by DIPG and other types of cancer (Chipumuro et al., 2014; Nagaraja et al., 2017).

## The role of lineage-specific transcriptional regulators in neurological cancers

Many transcription factors with well-characterised roles in neurogenesis and development of the nervous system have subsequently been identified as lineage-specific oncogenes and/or tumour suppressor genes in cancers of the central nervous system (CNS). This may illustrate the close relationship between normal developmental processes and tumourigenesis, and may reflect the influence of stem/progenitor cell positional identity on the response to oncogenic pathways. Indeed, stem and progenitors cells located in discrete brain regions and embedded in different supportive niches possess unique transcription factor codes from patterning processes (Azzarelli et al., 2015), as well as distinct growth requirements that could impinge on their susceptibility to specific oncogenic signals. Interrogating the expression and activity of lineage-specific transcriptional regulators in different contexts may shed light on the origin of nervous system cancers, as well as reveal potential new therapeutic vulnerabilities.

### Sox2

SOX2 is a prominent member of the sex-determining region (SRY) box 2 family of proteins that have wide-ranging roles in the developing embryo and in adult stem cells. Although eclipsed in recent years by its identification as a key pluripotency factor, SOX2 has also been extensively studied in the context of its important roles in nervous system development and adult NSC activity. Likely reflecting these activities, SOX2 has emerged as a central player in neurological cancers.

SOX2 is often highly expressed in glioblastoma and its knockdown reduces proliferation and tumourigenicity in glioblastoma tumour-initiating cells (de la Rocha et al., 2014; Gangemi et al., 2009; Garros-Regulez et al., 2016). Mirroring its function in the maintenance of normal NSCs, SOX2 appears to act within a transcriptional network to propagate glioma-initiating properties and, therefore, acts as a driver of cancer stem cell-like behaviour. A combination of the transcriptional regulators Sox2, Olig2 and Zeb1 is robustly expressed in genetically diverse glioblastomas and is sufficient to transform astrocytes that have lost tumour suppressor gene pathways (Singh et al., 2017). Moreover, the possible role of SOX2 in driving gliomagenesis may also reflect its remarkable ability to facilitate active dedifferentiation of more mature cell types, e.g. in reprogramming of fibroblasts to induced pluripotent stem cells (Takahashi et al., 2007) and to NSC-like cells (Lujan et al., 2012). Forced expression of SOX2 in cooperation with FOXG1, another component of the fibroblast-to-NSC reprogramming cocktail that has also been implicated in glioblastoma, can impose a dedifferentiation programme on astrocytes that results in reactivation of cell division and acquisition of NSC-like characteristics (Bulstrode et al., 2017). SOX2 expression also indicates a potential role in the aetiology of paediatric tumours, including DIPG (Ballester et al., 2013) and SHH-type medulloblastoma (Vanner et al., 2014). Other SOX family members have been shown to have various roles as oncogenes and tumour suppressor genes in a variety of CNS tumours (de la Rocha et al., 2014), and it seems reasonable to speculate that their roles reflect a subversion of their normal developmental functions. Hence, better characterisation of these normal functions may reveal additional treatment vulnerabilities.

### bHLH proneural transcriptional regulators

The main functions of proneural basic helix-loop-helix (bHLH) transcription factors are to specify cell fate, to regulate NSC proliferation, and to drive neuronal differentiation during embryonic and postnatal development (Bertrand et al., 2002; Imayoshi and Kageyama, 2014). Although mutations in proneural bHLHs have not been consistently found in tumour samples, their expression is altered in several neural and endocrine cancers, suggesting that proneural proteins might play important roles in cancer initiation and maintenance (Huang et al., 2014) (Table 2). In addition, non-tissue-specific bHLH regulators, such as ID and HES proteins, have also been implicated in regulating tumourigenesis (Lasorella et al., 2014; Sang et al., 2010). The potential involvement of proneural factors in tumourigenesis, and in particular lineage-specific factors, such as ASCL1, OLIG2 and ATOH1, described below and in Table 2, again points to retention and subversion of transcriptional networks found in their normal counterpart cells.

**Table 2.**
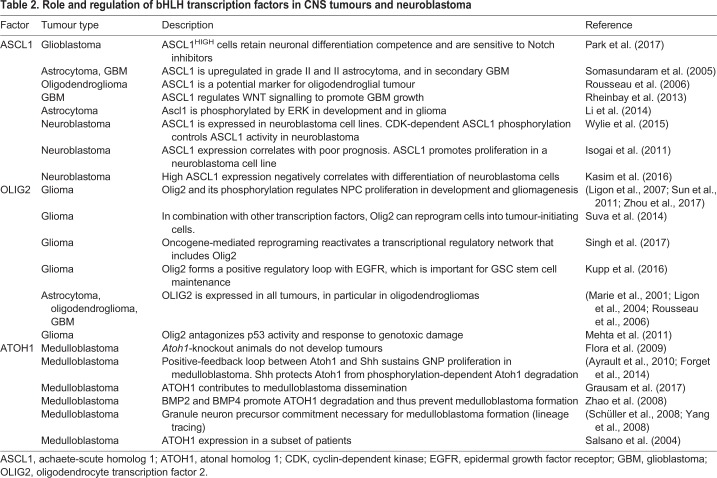
**Role and regulation of bHLH transcription factors in CNS tumours and neuroblastoma**

#### ASCL1 in gliomagenesis

During embryonic and postnatal development, ASCL1 plays an important role in the regulation of NSCs and oligodendrocyte precursors (Parras et al., 2004; Raposo et al., 2015). ASCL1 is frequently expressed in malignant brain tumours, including oliogdendroglioma, diffuse astrocytoma and proneural type glioblastoma, as well as in primary glioblastoma and lower grade gliomas (Rheinbay et al., 2013; Rousseau et al., 2006; Somasundaram et al., 2005). This expression may be a reflection of ASCL1 expression in the tumour cell of origin, as well as its functional role in neurogenesis.

ASCL1 expression is maintained in NSCs and glioma stem cells in culture, where it is essential for their proliferation and self-renewal in part through activation of Wnt signalling (Raposo et al., 2015; Rheinbay et al., 2013). However, the specific level of ASCL1, i.e. high versus low, does not strictly correlate with proliferative properties (Park et al., 2017). Instead, phenotypic differences that depend on the expression level of ASCL1 are more likely to emerge when cells undergo differentiation or in tumourigenic assays. Glioma stem cells derived from individuals with high ASCL1 expression remained more competent to undergo terminal neuronal differentiation in response to Notch inhibition compared with glioma stem cells expressing low ASCL1 (Park et al., 2017). As high ASCL1 expression in these patients apparently correlates with better clinical outcome, this has led to the suggestion of using ASCL1-based patient stratification to identify a subgroup of patients that can be effectively treated with Notch inhibitors to bring about ASCL1-mediated differentiation (Park et al., 2017). Previous attempts to differentiate glioma stem cells as a way to lock them permanently out of cell cycle have not been successful, as cells rapidly re-enter a proliferative state when treatment is removed and permanent epigenetic modifications indicative of stable differentiation are not evident (Carén et al., 2015).

Treatment of glioma cells with bone morphogenetic protein (BMP) causes cells to enter a state of reversible quiescence characterised by astrocytic marker expression (Carén et al., 2015; Martynoga et al., 2013; Park et al., 2017). In contrast, ASCL1-driven neuronal differentiation may lock cells in a post-mitotic state, re-imposing a normal developmental trajectory. Ascl1 has been shown to have roles in stem cell quiescence, as well as in stem/progenitor cell proliferation and differentiation during development and in adulthood, and it would be interesting to understand more about how these potentially opposing activities are controlled in normal development and subverted in cancer (Carén et al., 2015; Martynoga et al., 2013; Urbán et al., 2016).

The many roles of ASCL1 in stem/progenitor regulation indicate that its activity must be tightly controlled and, indeed, post-translational regulation by phosphorylation has been described. For example, multi-site phosphorylation by extracellular signal-regulated kinase ERK biases Ascl1-positive progenitors towards a proliferative glial program responsible for astrocytoma initiation (Li et al., 2014). Moreover, ASCL1 is highly phosphorylated by cyclin-dependent kinase (CDK) during embryonic development and neuronal reprogramming. In these contexts, phosphorylation of ASCL1 restrains its ability to promote differentiation, an effect analogous to that described for other proneural bHLHs in development and cancer (Ali et al., 2014; Azzarelli et al., 2017). In the future, it will be important to explore the integration of ERK and CDK-mediated ASCL1 phosphoregulation in glioma initiation and maintenance, and to explore whether inhibition of ASCL1 phosphorylation is a rational strategy with which to decrease tumourigenicity by potentiating glioma stem cell differentiation. In addition to a role in brain cancer biology, ASCL1 is expressed in various neuroendocrine tumours of the lung (Borromeo et al., 2016; Jiang et al., 2009), prostate and intestine, and in neuroblastoma (Isogai et al., 2011; Kasim et al., 2016; Wylie et al., 2015), indicating a potentially more widespread role in tumourigenesis (see Table 2).

#### OLIG2 in gliomagenesis

The transcriptional regulator OLIG2 cannot strictly be considered a ‘proneural’ transcription factor, since its main function is to induce gliogenesis and inhibit neurogenesis in oligodendrocyte precursor cells, although it also plays a role in motor neuron specification in the spinal cord (Lu et al., 2002; Novitch et al., 2001; Takebayashi et al., 2002). Potentially acting as a lineage-specific oncogene, OLIG2 is expressed in all cases of diffuse paediatric and adult human gliomas regardless of grade (Ligon et al., 2004; Marie et al., 2001). OLIG2 is required for proliferation of multipotent neural progenitors and for glioma formation in a mouse model of gliomagenesis and it is also expressed in replicating oligodendrocyte precursor cells where it cooperates with ASCL1 to specify oligodendrocytes (Ligon et al., 2007; Parras et al., 2004). In these contexts, its activity is controlled by phosphorylation of three specific serines, which results in sustained progenitor proliferation and glioma stem cell propagation, in part through repression of the CDK inhibitor CDKN1A (Mehta et al., 2011; Sun et al., 2011). More recently, the kinases responsible for OLIG2 phosphorylation have been identified and targeted by small molecule inhibitors that reduce gliomagenesis and increase survival in a BRAFV600E mouse model of paediatric glioma (Zhou et al., 2017). Importantly, OLIG2 phosphorylation is involved in a positive regulatory loop with receptor tyrosine kinases such as EGFR, which is essential for glioma stem cell maintenance *in vitro* (Kupp et al., 2016).

In addition to the roles of OLIG2 in glioma stem cell growth, tumour progression and differentiation, a role in tumour initiation, e.g. by promoting cell fate reprogramming of more differentiated cell types into stem-like cancer cells, is possible. Combined induction of three transcription factors (POU3F2, SOX2, SALL2) with OLIG2 (but not with ASCL1) in differentiated glioblastoma generates cells capable of initiating tumours with high efficiency (Suvà et al., 2014). Moreover, Olig2 has been recently identified as a key component of the transcriptional regulatory network activated upon combination of tumour suppressor and oncogene mutations in astrocytes (Singh et al., 2017). This demonstrates that oncogene-mediated dedifferentiation/reprogramming could directly reactivate these lineage-specific stem/progenitor genes.

#### ATOH1 in medulloblastoma

ATOH1 is expressed in granule neuron precursors of the postnatal cerebellum and is highly expressed in SHH-type medulloblastomas (Table 2) (Salsano et al., 2004). Although Atoh1 overexpression is not sufficient to drive full tumourigenesis, commitment to the Atoh1-positive granule neuron precursor lineage is an essential requirement for medulloblastoma formation (Schüller et al., 2008), indicating essential crosstalk between developmental and tumourigenic programmes. Atoh1 activity works to drive medulloblastoma only in the context of underlying Shh mutations, whereas Atoh1 loss of function prevents medulloblastoma formation due to decreased granule neuron precursor proliferation and impaired Shh signalling (Flora et al., 2009; Grausam et al., 2017). Moreover, positive feedback exists between Atoh1 and Shh, whereby Atoh1 maintains granule neuron precursors in a Shh-responsive state, in part through the activation of the Shh target Gli2; in turn Shh sustains Atoh1 expression and granule neuron precursor proliferation (Ayrault et al., 2010; Flora et al., 2009). Thus, Atoh1 function in granule neuron precursors and its interaction with Shh signalling represents the best example of how lineage-specific regulatory pathways result in selective vulnerabilities to specific oncogenic mutations.

Regulation of Atoh1 protein expression and stability is crucial for lineage progression and granule neuron precursor differentiation; Atoh1 destabilization and degradation, which coincides with NeuroD1 upregulation, is a key requirement for progression down the granule neuron lineage (Butts et al., 2014). Thus, proliferating Atoh1-positive granule neuron precursors in medulloblastoma may be locked in a pro-tumourigenic state resulting from a failure to properly differentiate due to sustained levels of Atoh1. The mechanisms that control Atoh1 stability are beginning to be uncovered, revealing a crucial role for phosphorylation-mediated degradation and for components of the BMP signalling pathway (Forget et al., 2014; Zhao et al., 2008). In common with the regulation of other bHLH proneural genes (Ali et al., 2011, 2014; Azzarelli et al., 2017; Hardwick and Philpott, 2015; Hindley et al., 2012), additional phosphorylation events potentially mediated by CDKs may play a more widespread role in controlling Atoh1 activity in both normal granule neuron precursors and in medulloblastoma.

## Conclusions

Much attention has been paid to the unpredictable heterogeneity of brain tumours and their aggressive growth characteristics, which have been used to explain their general resistance to treatment (Ellis et al., 2015; Gajjar et al., 2014). However, what is now emerging is a picture of cell behaviour that is far from chaotic. Instead, recent work suggests that, even though tumour cells have widespread genetic alterations, they may retain predictable behaviours that echo the proliferation and differentiation programmes from earlier times in development, and/or those seen in the context of adult stem/progenitor-based homeostasis or injury response that recapitulate these developmental programmes (Lan et al., 2017; Tirosh et al., 2016). Recapitulation of developmental phenotypes is even stronger in many paediatric tumours, where heterogeneity often arises from different behaviours of distinct developmental precursors.

Indicative of an underlying hijacking of neurodevelopment programmes, a number of transcriptional regulators of developmental neurogenesis act as lineage-specific oncogenes in CNS cancers. Genes such as *ASCL1* and *ATOH1* are predominantly expressed in embryonic and postnatal neurological development, and make appealing targets for therapy, although attention should be paid to the residual function of these genes in the small population of adult NSCs (Urbán et al., 2016) and to potential roles in regeneration after injury. Although transcription factors generally make poor drug targets, the manipulation of post-translational modifications of proneural proteins is emerging as a potential way to control the transcriptional activity of these genes (Ali et al., 2014; Wylie et al., 2015), and points to the existence of vulnerabilities that are specific to aberrant progenitor cells.

Although killing cancer cells is almost always the goal of current therapies, if CNS cancers arise from a dysregulation or stalling of developmental processes, an exciting possibility emerges that reactivation of a programme of differentiation will ultimately generate post-mitotic cells, and thus halt tumour growth (Wang and Chen, 2008). This idea, referred to as differentiation therapy, has been long discussed, and may become a reality as we begin to better understand what controls both lineage progression and the balance between proliferation and differentiation in normal and malignant tissues. In particular, targeting multi-site phosphorylation of the proneural proteins that act as master regulators of proliferation and differentiation throughout the CNS should be further explored as a potential new way to tip the balance of stem and progenitor cells in favour of the post-mitotic differentiated state.

Beyond the promise and obvious challenges of targeting individual transcriptional networks, our understanding of how the wider epigenetic landscape influences fate choice, proliferation and differentiation is constantly improving. A clear goal is to use drugs that can influence the epigenome to change the fate and behaviour of cells in response to the endogenous transcriptional programmes, although the specificity of this approach *in vivo* remains to be tested fully. Another area in its infancy and yet to be explored fully in the CNS is the concept that changing the tumour microenvironment may lead to changes in behaviour of the tumour cells themselves; by manipulating the niche, we may shut down the tumour stem cell-like programme. Such a possibility has been suggested by work in other tissues but remains open for investigation in the nervous system (Burger and Peled, 2009; Calabrese et al., 2007; Tauriello et al., 2018).

Overall, it is clear that our understanding of the behaviour of brain tumour cells is growing rapidly and will be further enhanced by understanding how aberrant tumour cell behaviour often represents a reversion to a dysregulated developmental phenotype. If we are to further understand this phenomenon and to exploit emerging vulnerabilities that result in either the death or differentiation of tumour cells, we need to have more engagement between developmental biologists and cancer biologists. After all, in many ways cancer is ‘development gone wrong’, so developmental biologists are as well placed as any scientists to help understand and treat these devastating diseases.
